# Blennorrhœa

**Published:** 1891-12

**Authors:** James T. Jelks

**Affiliations:** Hot Springs, Ark. Formerly a member of State Medical Society of Georgia; a member of Arkansas State Medical Society and Medical Association; late Professor of Surgical Diseases of the Genito-Urinary Organs and Venereal Diseases, in College of Physicians and Surgeons, Chicago, Illinois


### BLENNORRHŒA.

ABSTRACT OF PAPER READ BEFORE THE MISSISSIPPI VALLEY MEDICAL ASSOCIATION BY DR. JAMES T. JELKS, OF HOT SPRINGS, ARK. Formerly a member of State Medical Society of Georgia; a member of Arkansas State Medical Society and Medical Association; late Professor of Surgical Diseases of the Genito-Urinary Organs and Venereal Diseases, in College of Physicians and Surgeons, Chicago, Illinois.

Ricord's conclusion that gonorrhœa was a non-virulent disease has been relegated to the rear by finding the gonococcus of Neisser. All efforts to produce a blennorrhœa with ordinary pus have failed. Every effort to produce the disease with the discharge of the first stage of the disease where the pus corpuscles are few succeeds.

The identity of blennorrhœa neonatorum and ordinary gonorrhœal disease has been established.

Neisser demonstrated the gonococcus in 1879. His conclusions were confirmed in 1880 by Bokai and Finkelstein. These men cultivated the gonococcus, and with the pure cultures produced typical urethritis.

Bockhardt, in 1883, reported the results of his examinations. In 258 cases he invariably found the gonococcus. He likewise cultivated it, and with the culture produced blennorhœa. Keyser in sixty cases found the same germ.

In 1884 Zweifel reported that only that pus which contained the gonococcus of Neisser could produce blennorrhœa neonatorum.

Bumm confirmed these statements of Zweifel.

Sternberg, in 1884, disputed these statements of Bumm, Neisser, Bokai, Finkelstein, Bockhardt, Zweifel, Willands and many others. With Sternberg we find Sanger, Frankel, Gervanin, M. von Zeisel and Lustgarten and Mannaberg.

In 1886 Bumm again wrote concerning the coccus of Neisser. In numerous cultivation experiments he demonstrated the presence of this coccus, and with pure cultures of the 2d and 29th generation produced, in two women, typical urethritis and in the discharge was found the gonococcus. This, then, is a demonstration that urethritis is a specific disease.

This specific disease of the urethra is in a large majority of cases limited to the anterior portion of the canal. When the posterior is involved it should be looked upon as a complication and treated as such by cessation of all treatment of the anterior urethra and rest in bed.

You are all familiar with Fournier's celebrated statement as to the sources of infection. Out of 387 cases the disease was acquired from regular prostitutes twelve times; and in 375 cases from clandestine prostitution!

Bumm's experiments with pure cultures in the eyes of rabbits has enabled us to follow the gonococcus in its travels. First they multiply upon the epithelium. In a few days they penetrate this layer of the mucous membrane—and henceforth propagate within and upon the papillary layer. This process of sub-epithelial multiplication of the gonococcus for the first two or three weeks of a blennorrhœa is one of great importance to us; and not until the decline of the disease is this process changed to one of surface multiplication. This is a very important fact for us, and explains why all of our efforts to jugulate the disease have failed. It is true that surgeons, years ago, used strong solutions of silver nitrate, twenty to forty grs. to the oz. to abort the disease, and sometimes succeeded; but when they did it was because this strong remedy destroyed the epithelium, and hence reached the coccus beneath it; but the remedy was worse than the disease.

### TREATMENT.

I will not go over all the old remedies and modes of treating blennorrhœa, but call your attention to Prof. Stilling's experiments with Merk's Pyoktanin. He used it successfully in blennorrhoea neonatorum. Last winter I heard Dr. Holtz read a paper before the Chicago Medical Society giving his experience with pyoktanin in gonorrhœal ophthalmia.

By reason of the fact that pyoktanin penetrates the epithelial covering and into the papillary layer, it occurred to me that this was the remedy for blennorrhœa; and I once instructed the attending staff in the Genito-Urinary Department of the West Side Free Dispensary of Chicago to use it.

Upon my return home I commenced its use in my private practice. Some of the cases have gotton well with marvellous rapidity, a few in twenty-four to forty-eight hours. Again others have obtained no benefit from the remedy. I am not able to tell why this should be.

On June 13th, 1891, Dr. George Wiley Broome, of St. Louis, Mo., read a paper before the St. Louis Medical Society, wherein he advocated the use of pyoktanin in blennorrhœa. His method was to insufflate the dry powder. My plan has been to use a saturated solution as an injection, retaining the fluid for five or ten minutes and by pressure forcing it down to the isthmus.

I am sure that the treatment of the future will lie in getting some remedy which has the power of penetrating the tissues and hence reach the disease germs.

---

ASSOCIATION OF AMERICAN MEDICAL COLLEGES.—The Association of American Medical Colleges held its second annual session recently in Washington, when a permanent constitution and by-laws were formally adopted. The object of the Association is to raise the standard of education, both general and technical, required for admission to the medical profession. Colleges becoming members of the Association undertake to demand of candidates for the degree of M. D. that they shall attend at least three full courses of medical lectures of not less than six months each, no two of these courses being in the same year. They must further require candidates for admission to the courses to pass an entrance examination on a scheme prepared by Dr. W. Osler and Dr. Millard, which includes writing "legibly and correctly" an English composition of not less than 200 words, the translation of an easy piece of Latin prose, higher arithmetic or the elements of algebra, and elementary physics. Only graduates or matriculated students of recognized colleges of literature, science, or arts are to be exempted from this examination, which is to come into force in 1892. On the whole, the requirements can hardly be called oppressive, but they appear to have been sufficient to damp the zeal of more than one of the colleges which had shown a disposition to join the Association.—*Exchange.*

